# Alcoholic Beverage Preference and Dietary Habits in Elderly across Europe: Analyses within the Consortium on Health and Ageing: Network of Cohorts in Europe and the United States (CHANCES) Project

**DOI:** 10.1371/journal.pone.0161603

**Published:** 2016-08-22

**Authors:** Diewertje Sluik, Nicole Jankovic, Mark G. O’Doherty, Anouk Geelen, Ben Schöttker, Olov Rolandsson, Jessica C. Kiefte-de Jong, Jean Ferrieres, Christina Bamia, Heidi P. Fransen, Jolanda M. A. Boer, Sture Eriksson, Begoña Martínez, José María Huerta, Daan Kromhout, Lisette C. P. G. M. de Groot, Oscar H. Franco, Antonia Trichopoulou, Paolo Boffetta, Frank Kee, Edith J. M. Feskens

**Affiliations:** 1 Division of Human Nutrition, Wageningen University, Wageningen, the Netherlands; 2 Centre of Clinical Epidemiology, Institute for Medical Informatics, Biometry and Epidemiology, Faculty of Medicine, University Duisburg-Essen, Essen, Germany; 3 UKCRC Centre of Excellence for Public Health, Queens University Belfast, Belfast, Northern Ireland; 4 Division of Clinical Epidemiology and Aging Research, German Cancer Research Center (DKFZ), Heidelberg, Germany; 5 Institute of Health Care and Social Sciences, FOM University, Essen, Germany; 6 Department of Public Health and Clinical Medicine, Family Medicine, Umeå University, Umeå, Sweden; 7 Global Public Health, Leiden University College, the Hague, the Netherlands; 8 Department of Epidemiology, Erasmus Medical Center, Rotterdam, the Netherlands; 9 Department of Cardiology, Toulouse University School of Medicine, Toulouse, France; 10 Hellenic Health Foundation, Athens, Greece; 11 WHO Collaborating Center for Nutrition and Health, Unit of Nutritional Epidemiology and Nutrition in Public Health, Department of Hygiene, Epidemiology and Medical Statistics, University of Athens Medical School, Athens, Greece; 12 Julius Center for Health Sciences and Primary Care, University Medical Center Utrecht, Utrecht, the Netherlands; 13 National Institute for Public Health and the Environment (RIVM), Bilthoven, the Netherlands; 14 Escuela Andaluza de Salud Pública, Instituto de Investigación Biosanitaria ibs.Granada, Hospitales Universitaios de Granada/Universidad de Granada, Granada, Spain; 15 CIBER de Epidemiología y Salud Pública (CIBERESP), Madrid, Spain; 16 Department of Epidemiology, Murcia Regional Health Council, IMIB-Arrixaca, Murcia, Spain; 17 Icahn School of Medicine, Mount Sinai School of Medicine, New York, United States of America; Medizinische Fakultat der RWTH Aachen, GERMANY

## Abstract

**Introduction:**

The differential associations of beer, wine, and spirit consumption on cardiovascular risk found in observational studies may be confounded by diet. We described and compared dietary intake and diet quality according to alcoholic beverage preference in European elderly.

**Methods:**

From the Consortium on Health and Ageing: Network of Cohorts in Europe and the United States (CHANCES), seven European cohorts were included, i.e. four sub-cohorts from EPIC-Elderly, the SENECA Study, the Zutphen Elderly Study, and the Rotterdam Study. Harmonized data of 29,423 elderly participants from 14 European countries were analyzed. Baseline data on consumption of beer, wine, and spirits, and dietary intake were collected with questionnaires. Diet quality was assessed using the Healthy Diet Indicator (HDI). Intakes and scores across categories of alcoholic beverage preference (beer, wine, spirit, no preference, non-consumers) were adjusted for age, sex, socio-economic status, self-reported prevalent diseases, and lifestyle factors. Cohort-specific mean intakes and scores were calculated as well as weighted means combining all cohorts.

**Results:**

In 5 of 7 cohorts, persons with a wine preference formed the largest group. After multivariate adjustment, persons with a wine preference tended to have a higher HDI score and intake of healthy foods in most cohorts, but differences were small. The weighted estimates of all cohorts combined revealed that non-consumers had the highest fruit and vegetable intake, followed by wine consumers. Non-consumers and persons with no specific preference had a higher HDI score, spirit consumers the lowest. However, overall diet quality as measured by HDI did not differ greatly across alcoholic beverage preference categories.

**Discussion:**

This study using harmonized data from ~30,000 elderly from 14 European countries showed that, after multivariate adjustment, dietary habits and diet quality did not differ greatly according to alcoholic beverage preference.

## Introduction

The relationship between alcohol and cardiovascular diseases (CVD) is assumed to be J-shaped [1]. Several studies have reported differential effects of alcoholic beverages in respect of the protection against CVD, mostly in favor of wine consumption [2–4]. However, a recent meta-analysis indicated that moderate consumption of both wine and beer could reduce the risk of CVD [1]. It may be that the preference for a specific alcoholic beverage is related to the overall dietary pattern and the confounding effect of diet may partially explain the difference in risk. However, not many studies into health effects of alcoholic beverage consumption have taken diet into account.

The study of Tjønneland *et al*. was one of the first to investigate the association between alcoholic beverage intake and diet and received much attention. In a sample of ~50,000 Danish men and women, they observed that wine consumption was related to a healthier diet: wine consumers had higher intakes of fruit, fish, vegetables, and olive oil in comparison to the consumption of other alcoholic beverages [5]. Several studies in other European countries and the U.S. have followed since. A systematic literature review summarizing these studies showed that people with a beer preference generally have a lower diet quality. For wine consumers, a difference between Western and Mediterranean study populations was observed: a wine preference was related to a healthier diet in Northern-Europe and the United States (US) whereas for most Southern-European countries, no such relation was seen [6]. Furthermore, persons with a higher consumption frequency, i.e. number of drinking days, have shown to display a healthier diet, but diet quality decreased with higher absolute amounts of alcohol [7].

Previous studies regarding alcoholic beverage preference and diet showed a wide variety of different associations across study populations and countries. This can be due to the fact that they did not use a uniform definition of alcoholic beverage preference: some used absolute intake of beer, wine, or spirits [8, 9], others defined preference as the drink that accounted for a certain percentage (>50%, >70%, >75%) of the total alcohol consumption [10, 11], or alcohol preference was poorly assessed with one question only [12]. Furthermore, a wide range of dietary factors was included: from nutrients, to foods and dietary patterns. Since people consume total diets instead of single dietary components it is most informative to study dietary patterns and diet quality. Therefore, the present cross-sectional study aimed to describe and compare dietary habits and diet quality with alcoholic beverage preference across several European countries using harmonized data. By using a uniform definition of alcoholic beverage preference and standardized measures of dietary intake, between-country comparisons can be made. Furthermore, by focusing on dietary components as well as overall diet quality, we study the whole spectrum of diet.

## Materials and Methods

### Study design and population

The Consortium on Health and Ageing: Network of Cohorts in Europe and the United States (CHANCES) project is a multi-country study aiming at the harmonization of data from prospective cohort studies in Europe and the US in order to produce evidence on ageing-related health characteristics and determinants of healthy ageing among the elderly in these countries [13]. The CHANCES project includes cohorts from 14 studies across Europe and the USA. In most CHANCES cohorts, elderly are defined as those who were 60 years or older at recruitment. The CHANCES project as a whole has received ethical approval by the Hellenic Health Foundation Committee on Bioethics (HHFCB). We did not have any access to personal information regarding the participants included in this paper. All data that have been analyzed are based on the CHANCES harmonized variables and are completely anonymized [14]. In the individual cohorts, all participants signed informed consent for the original studies. Commonly, this was a general statement with no mention to detailed type of studies or specific objectives to be dealt in the future. With this, the participants acknowledged that the information and material they provided was to be used in future research, including the current study.

Within the CHANCES consortium, seven European cohorts were found eligible to answer our study question based upon availability of data and logistical reasons: the European Prospective Investigation into Cancer and Nutrition (EPIC)–Elderly study centers of Spain, the Netherlands, Greece, and Sweden [15], the Rotterdam Study [16], the Survey Europe on Nutrition in the Elderly: a Concerted Action (SENECA) study [17], and the Zutphen Elderly Study [18]. EPIC is a multicenter, prospective cohort study, where 519,978 apparently healthy volunteers from 23 centers in 10 European countries were recruited between 1992 and 2000. The EPIC-Elderly cohort includes all participants aged 60 or over at recruitment. The EPIC study has received ethical approval by the IARC Ethical Review Committee. The Rotterdam Study is a prospective cohort study among 7,983 persons aged 55 years and over who live in a defined geographic area in Rotterdam, the Netherlands. Baseline examinations were performed between 1990 and 1993. The Rotterdam Study has been approved by the Medical Ethics Committee of the Erasmus MC and by the Ministry of Health, Welfare and Sport of the Netherlands, implementing the Wet Bevolkingsonderzoek: ERGO (Population Studies Act: Rotterdam Study). SENECA is a prospective mixed cross-sectional and longitudinal study that recruited about 2,000 individuals born between 1913 and 1918 from 12 European countries in 1988 to 1989. Ethical approval was obtained from the local ethical committees in all 19 included towns of Hamme in Belgium, Roskilde in Denmark, Chateau Renault-Amboise, Haguenau and Romans in France, Anogia-Archanes and Markopoulou in Greece, Monor in Hungary, Padua and Fara Sabina Magliano Sabina-Poggio Mirteto in Italy, Culemborg in the Netherlands, Elverum in Norway, Marki in Poland, Coimbra and Vila Franca de Xira in Portugal, Betanzos in Spain, and Yverdon, and Burgdorf and Bellinzona in Switzerland. The Zutphen Elderly Study is a prospective population-based cohort study including a total sample of 876 men aged 65 to 84 years living in the city of Zutphen, the Netherlands. The Zutphen Elderly Study was approved by the Medical Ethics Committee of the University of Leiden in 1985 and 1990 and by the Medical Ethics Committee of the Netherlands Organization for Applied Scientific Research in 1995 and 2000.

Within the cohorts that were eligible for the present study, analyses were conducted upon all subjects without missing data on alcohol and dietary intake. For some cohorts, the confounding variables education, employment status, physical activity, and smoking were missing, not available or not applicable. In order to treat all subjects from each cohort equally, subjects with missing values for these covariates were not excluded. For categorical variables, a joint indicator variable for missing values was composed and for continuous variables the median value in the respective cohort was imputed. Furthermore, subjects with self-reported prevalent diabetes at baseline were excluded for analysis, since their diagnosis may have influenced their diet and alcohol consumption. The numbers in the SENECA Study did not allow comparisons between alcoholic beverage preference categories and diet by country. Therefore, the cohort was divided into SENECA Northern Europe, including the Netherlands, Belgium, Denmark, Hungary, Norway, Switzerland, and Poland and SENECA Southern Europe, including France, Greece, Italy, Portugal, and Spain. The analytical sample sizes were n = 4565 for EPIC-Elderly Spain, n = 6547 for EPIC-Elderly the Netherlands, n = 8418 for EPIC-Elderly Greece, n = 3263 for EPIC-Elderly Sweden, n = 3571 for the Rotterdam Study, n = 1069 for SENECA Northern Europe, n = 1169 for SENECA Southern Europe, and finally n = 794 for the Zutphen Elderly Study.

### Data assessment and harmonization

Data in the CHANCES project have been collected within the framework of independent cohort studies, with different protocols for data collection and distinct original research foci. Data harmonization was a major task of the project and the data harmonization and conversion rules of the CHANCES project have been described elsewhere [14]. Data standardization and harmonization procedures were largely based upon the experience from the MORGAM project [19] and previous experiences of project partners. Data assessment procedures included examination of availability and comparability of cohort data, questionnaires and measurement procedures used in the individual cohorts, methods for collection of data on health outcomes and of blood samples.

### Dietary assessment

Different dietary assessment methods were used across cohorts; furthermore, the total number of food items, reference periods, and interview-derived or self-reported dietary assessments differed. Translation of foods into nutrients was performed using cohort-specific food composition tables. Jankovic *et al*. have given a detailed overview of the dietary assessment methods of the CHANCES cohorts in a supplementary table [20]. EPIC-Elderly the Netherlands, Greece, and Sweden and the Rotterdam Study applied a validated food frequency questionnaire [16, 21]. EPIC-Elderly Spain, SENECA and the Zutphen Elderly Study used a validated dietary history method [22–24]. Within the CHANCES project, standardized and harmonized variables of food groups and nutrients were created for all cohorts.

### Alcoholic beverage preference

If not already defined in the cohorts, average daily alcohol consumption in grams was estimated by adding the amounts of pure alcohol found in each standard drink or cohort specific size for beer, wine, and spirits. To ensure comparability across cohorts, a conversion rule was applied using standardized portion sizes (330 ml for a bottle of beer, 175 ml for a glass of wine, and 25 ml for a shot of spirit) and alcohol percentages in beer (4.5%), wine (12%), and spirits (37.5%) [14]. As defined in previous studies [25, 26], a person was classified as having a preference for beer, wine, or spirits, when the alcohol consumption from the respective drink comprised 70% or more of the total alcohol consumption. When the average alcohol consumption from either beer, wine, or spirits did not reach 70% of the total alcohol consumption, a person was classified as having no preference. Persons who reported not to consume any alcohol were classified as non-consumers.

### Healthy Diet Indicator (HDI)

Huijbregts *et al*. developed a dichotomous Healthy Diet Indicator (HDI) score based upon the 1990 WHO dietary guidelines [27]. This score was updated to a continuous scoring system using the 2003 WHO dietary guidelines by Jankovic *et al*. [20]. The current HDI score reflects the most current WHO guidelines on diet for the prevention of chronic diseases. The score includes 6 nutrients and 1 food group of the 14 WHO guideline goals, which were available for all included cohorts: percentages of energy intake from saturated fatty acids, polyunsaturated fatty acids, mono- and disaccharides, and protein; and intakes of cholesterol (mg/day), fruits and vegetables (g/day), and either total dietary fiber or non-starch polysaccharides (g/day). For the 7 HDI components, the maximum score of 10 points was allocated if the intake was in accordance with the WHO guidelines. The maximum total HDI score was 70 if all guidelines were met [20].

### Covariate assessment

Socio-demographic factors including highest level of educational attainment and employment status, and lifestyle factors including smoking behavior and physical activity, and disease history of coronary heart disease (CHD) and cancer were assessed by self-administered questionnaires or by interviews. Weight and height were measured in all cohorts, and total and HDL cholesterol were determined from blood samples. These variables were also standardized and harmonized within the CHANCES framework.

### Statistical analysis

All statistical analyses were performed using SAS, version 9.3, software (SAS Institute, Inc., Cary, North Carolina). By cohort, adjusted means and standard errors of nutrient and food group intakes and HDI scores across alcoholic beverage preference categories were calculated using generalized linear models (analysis of covariance). Adjusted means were calculated by fitting a regression model using the variable of interest as the dependent variable and the covariates as independent variables. Combining all cohorts, inverse-variance weighted means were calculated. Based on a priori knowledge, factors that were associated with both alcoholic beverage preference and dietary habits were selected as potential confounders. Adjustments were made for age, gender (not applicable for Zutphen Elderly Study, since they included men only), education (primary or less (low), more than primary but less than college or university (middle), college or university (high)), employment status (not applicable for Zutphen Elderly Study and SENECA, where participants were all retirees, and not assessed in EPIC-Elderly Spain; full-time or part-time employment and not of pensionable age, self-employed, housewife and not of pensionable age, pensionable age and still working, pensionable age and not working, stopped work before retirement age due to poor health, unemployed and not of pensionable age), self-reported prevalent CHD (yes/no) or cancer (yes/no), smoking status (never, former, current), sports activity (hours per week; log-transformed; in the Rotterdam Study total physical activity measured at first follow-up was used; not available at time of analysis for EPIC-Elderly Sweden), moderate alcohol consumption (<0–12 gram/day; yes/no), and energy intake (kcal/day). To assess the robustness of the results, a sensitivity analysis was conducted where persons with self-reported prevalent diabetes were included in the analyses. Next to excluding persons with diabetes, a second sensitivity analysis further excluded persons with self-reported prevalent CHD or cancer from the main analyses. In addition, stratified analysis according to gender, BMI categories (<25 kg/m^2^ and ≥25 kg/m^2^), and categories of absolute alcohol consumption (>0–12 g/d and ≥12 g/d) were conducted.

## Results

### General characteristics

Table 1 shows the general characteristics of the elderly of the separate cohorts across categories of alcoholic beverage preference. In SENECA Southern Europe, the Rotterdam Study, EPIC-Elderly Spain, and EPIC-Elderly the Netherlands, persons with a wine preference formed the largest group, ranging from 43 to 58%. In the Zutphen Elderly Study, the group of spirit preference was largest with 43% and in EPIC-Elderly Sweden, the group with no preference with 41%. In EPIC-Elderly Greece, the group of persons with a wine preference and non-consumers were of almost equal size.

**Table 1:** General characteristics of elderly included in the EPIC-Elderly (Spain, the Netherlands, Greece, and Sweden), Rotterdam Study, SENECA (Northern Europe and Southern Europe), and the Zutphen Elderly Study across categories of alcoholic beverage preference.

	Beer preference	Wine preference	Spiritpreference	No preference	Non-consumers
**EPIC-Elderly Spain**					
N (%)	263 (6)	2140 (47)	55 (1)	277 (6)	1857 (40)
Age, years	62.5 (1.7)	62.4 (1.6)	64.3 (2.6)	62.4 (1.5)	62.6 (1.7)
Male, n (%)	105 (40)	1315 (61)	70 (13)	210 (76)	313 (17)
Alcohol, g/d	2.2 [0.9–6.8]	17.0 [4.9–36.7]	8.4 [1.8–19.7]	15.7 [6.2–32.3]	0 [0–0]
BMI, kg/m^2^	29.3 (3.8)	29.2 (3.8)	26.4 (3.9)	29.3 (3.7)	29.9 (4.4)
Sports, hrs/wk	0.5 (1.5)	0.7 (1.8)	1.0 (1.7)	0.6 (1.5)	0.5 (1.5)
Education, n (%)					
- Low	212 (81)	1794 (85)	221 (41)	255 (84)	1637 (90))
- Medium	18 (7)	183 (9)	278 (52)	27 (9)	100 (5)
- High	31 (12)	140 (7)	37 (7)	23 (8)	95 (5)
Smoking status, n (%)					
- Never	173 (66)	1193 (56)	196 (36)	107 (39)	1575 (85)
- Former	38 (14)	459 (21)	179 (33)	70 (25)	162 (9)
- Current	52 (20)	485 (23)	162 (30)	100 (36)	118 (6)
Self-reported medical history, n (%)					
- CHD	10 (4)	10 (4)	30 (6)	7 (3)	47 (3)
- Cancer	12 (5)	12 (5)	44 (8)	2 (1)	69 (4)
Moderate alcohol consumption, n (%)*	219 (83)	854 (40)	292 (54)	120 (43)	NA
**EPIC-Elderly the Netherlands**					
N (%)	127 (2)	3783 (58)	541 (8)	708 (11)	1388 (21)
Age, years	63.9 (2.6)	64.4 (2.8)	64.3 (2.6)	64.3 (2.8)	64.7 (2.8)
Male, n (%)	48 (38)	73 (2)	70 (13)	85 (12)	31 (2)
Alcohol, g/d	5.8 [1.2–18.3]	4.2 [1.2–12.3]	8.4 [1.8–19.7]	4.7 [1.5–12.4]	0 [0–0]
BMI, kg/m^2^	25.7 (4.1)	26.0 (3.8)	26.4 (3.9)	25.8 (3.5)	26.7 (4.6)
Sports, hrs/wk	1.2 (2.0)	1.2 (1.8)	1.0 (1.7)	1.2 (1.7)	0.8 (1.6)
Education, n (%)					
- Low	58 (46)	998 (26)	221 (41)	196 (28)	676 (49)
- Medium	62 (49)	2230 (59)	278 (52)	423 (60)	635 (46)
- High	7 (6)	541 (14)	37 (7)	83 (12)	69 (5)
Smoking status, n (%)					
- Never	21 (17)	1711 (45)	196 (36)	310 (44)	849 (62)
- Former	51 (40)	1460 (39)	179 (33)	269 (38)	311 (23)
- Current	55 (43)	601 (16)	162 (30)	124 (18)	220 (16)
Self-reported medical history, n (%)					
- CHD	6 (5)	132 (3)	30 (6)	33 (5)	87 (6)
- Cancer	5 (4)	282 (7)	44 (8)	44 (6)	107 (8)
Moderate alcohol consumption, n (%)*	81 (64)	2818 (74)	292 (54)	528 (75)	NA
**EPIC-Elderly Greece**					
N (%)	537 (6)	2715 (32)	378 (4)	2015 (24)	2773 (33)
Age, years	66.4 (4.4)	67.4 (4.6)	66.9 (4.4)	66.3 (4.5)	67.8 (4.5)
Male, n (%)	254 (47)	1245 (46)	279 (74)	1049 (52)	493 (18)
Alcohol, g/d	1.3 [0.6–8.2]	7.3 [1.2–16.2]	13.2 [4.4–22.1]	2.8 [1.3–12.7]	0 [0–0]
BMI, kg/m^2^	29.1 (4.7)	28.8 (4.4)	28.8 (4.3)	28.8 (4.3)	29.8 (5.0)
Sports, hrs/wk	0.2 (0.9)	0.3 (0.9)	0.2 (0.9)	0.4 (1.2)	0.2 (0.7)
Education, n (%)					
- Low	489 (92)	2494 (92)	346 (92)	1679 (84)	489 (95)
- Medium	24 (4)	147 (5)	18 (5)	196 (10)	103 (24)
- High	21 (4)	62 (2)	13 (3)	135 (7)	42 (2)
Smoking status, n (%)					
- Never	347 (65)	1746 (66)	142 (39)	1179 (61)	2322 (85)
- Former	112 (21)	554 (21)	116 (32)	437 (23)	257 (9)
- Current	65 (12)	340 (13)	105 (29)	315 (16)	149 (5)
Self-reported medical history, n (%)					
- CHD	39 (7)	157 (6)	30 (8)	100 (5)	165 (6)
- Cancer	23 (4)	91 (3)	18 (5)	64 (3)	111 (4)
Moderate alcohol consumption, n (%)*	454 (85)	1920 (71)	174 (46)	1472 (73)	NA
**EPIC-Elderly Sweden**					
N (%)	963 (30)	416 (13)	139 (4)	1329 (41)	416 (13)
Age, years	60.4 (1.4)	60.3 (0.9)	60.1 (1.2)	60.3 (1.0)	60.4 (1.3)
Male, n (%)	559 (58)	61 (15)	107 (77)	711 (54)	78 (19)
Alcohol, g/d	0.9 [0.3–2.7]	1.6 [0.1–3.3]	2.0 [0.2–2.4]	2.9 [0.4–5.4]	0 [0–0]
BMI, kg/m^2^	25.6 (3.8)	25.5 (3.7)	26.7 (5.1)	25.9 (4.1)	26.2 (4.6)
Sports, hrs/wk	N.A.	N.A.	N.A.	N.A.	N.A.
Education, n (%)					
- Low	544 (57)	184 (44)	97 (70)	675 (51)	283 (68)
- Medium	308 (32)	156 (38)	34 (24)	470 (36)	102 (25)
- High	103 (11)	74 (18)	6 (4)	169 (13)	30 97)
Smoking status, n (%)					
- Never	646 (69)	273 (67)	49 (35)	648 (50)	341 (84)
- Former	174 (19)	82 (20)	34 (24)	354 (28)	40 (10)
- Current	120 (13)	53 (13)	53 (38)	285 (22)	27 (7)
Self-reported medical history, n (%)					
- CHD	27 (3)	5 (1)	6 (4)	43 (3)	6 (1)
- Cancer	50 (5)	32 (8)	2 (1)	72 (5)	31 (7)
Moderate alcohol consumption, n (%)*	939 (98)	402 (97)	132 (95)	1260 (95)	NA
**Rotterdam Study**					
N (%)	209 (6)	1553 (43)	724 (20)	453 (13)	632 (18)
Age, years	62.9 (5.9)	65.2 (7.0)	66.6 (6.8)	64.5 (6.3)	66.8 (7.4)
Male, n (%)	184 (88)	260 (17)	516 (71)	327 (72)	139 (22)
Alcohol, g/d	10.8 [2.2–23.5]	2.7 [0.5–8.6]	18.7 [7.4–32.0]	11.4 [3.8–24.6]	0 [0–0]
BMI, kg/m^2^	25.6 (3.0)	26.4 (3.7)	26.4 (3.2)	25.8 (2.9)	26.5 (4.1)
Sports, hrs/wk	2565 (1124)	2662 (1109)	2472 (1061)	2598 (1141)	2428 (1209)
Education, n (%)					
- Low	65 (31)	488 (31)	186 (26)	103 (23)	277 (44)
- Medium	119 (57)	940 (61)	462 (64)	274 (60)	320 (51)
- High	25 (12)	125 (8)	76 (11)	76 (17)	35 (6)
Smoking status, n (%)					
- Never	21 (10)	700 (45)	98 (14)	82 (18)	315 (50)
- Former	126 (60)	572 (37)	401 (55)	275 (61)	200 (32)
- Current	62 (30)	280 (18)	223 (31)	96 (21)	115 (18)
Self-reported medical history, n (%)					
- CHD	32 (15)	132 (9)	115 (16)	51 (11)	84 (13)
- Cancer	15 (7)	109 (7)	64 (9)	36 (8)	64 (10)
Moderate alcohol consumption, n (%)*	125 (66)	1242 (80)	261 (36)	240 (53)	NA
**SENECA: Northern Europe**					
N (%)	140 (13)	312 (29)	104 (10)	112 (10)	401 (38)
Age, years	73.2 (1.7)	73.3 (1.9)	73.0 (1.8)	72.9 (1.7)	73.7 (1.9)
Male, n (%)	92 (66)	160 (51)	68 (65)	78 (70)	136 (33)
Alcohol, g/d	8.5 [3.0–19.0]	10.0 [3.0–20.0]	6.7 [1.0–13.0]	13.0 [5.5–23.5]	0 [0–0]
BMI, kg/m^2^	26.1 (3.8)	26.3 (4.0)	26.5 (3.5)	26.2 (4.0)	26.7 (4.8)
Sports, hrs/wk	0.4 (1.4)	0.9 (1.7)	0.6 (1.4)	0.9 (1.9)	0.4 (1.2)
Education, n (%)					
- Low	95 (68)	152 (49)	52 (50)	56 (50)	265 (66)
- Medium	38 (27)	109 (35)	46 (44)	45 (40)	109 (27)
- High	7 (5)	51 (16)	6 (6)	11 (10)	26 (6)
Smoking status, n (%)					
- Never	42 (30)	147 (47)	32 (31)	30 (27)	257 (64)
- Former	53 (38)	94 (30)	30 (29)	41 (37)	82 (20)
- Current	45 (32)	71 (23)	42 (40)	41 (37)	62 (15)
Self-reported medical history, n (%)					
- CHD	35 (25)	39 (13)	26 (25)	15 (13)	93 (23)
- Cancer	4 (3)	4 (1)	2 (2)	3 (3)	8 (2)
Moderate alcohol consumption, n (%)	80 (57)	192 (62)	77 (74)	54 (48)	NA
**SENECA: Southern Europe**					
N (%)	27 (2)	682 (58)	8 (1)	58 (5)	394 (34)
Age, years	73.2 (2.2)	73.3 (1.8)	72.9 (1.8)	73.4 (1.7)	73.4 (1.8)
Male, n (%)	16 (59)	410 (60)	3 (38)	48 (83)	107 (27)
Alcohol, g/d	2.0 [0.0–7.0]	17.0 [7.0–32.0]	1.5 [0.5–6.0]	16.0 [5.0–42.0]	0 [0–0]
BMI, kg/m^2^	27.4 (5.0)	26.8 (4.4)	25.1 (4.2)	27.4 (3.7)	27.0 (4.5)
Sports, hrs/wk	0.4 (1.3)	0.5 (1.3)	0.3 (0.5)	0.5 (1.5)	0.1 (0.7)
Education, n (%)					
- Low	21 (78)	476 (70)	4 (50)	38 (66)	323 (82)
- Medium	6 (22)	151 (22)	4 (50)	13 (22)	53 (13)
- High	0 (0)	54 (8)	0 (0)	7 (12)	15 (4)
Smoking status, n (%)					
- Never	12 (44)	342 (50)	3 (38)	16 (28)	297 (75)
- Former	11 (41)	223 (33)	2 (25)	27 (47)	76 (19)
- Current	4 (15)	117 (17)	3 (38)	15 (26)	21 (5)
Self-reported medical history, n (%)					
- CHD	1 (4)	90 (13)	0 (0)	8 (14)	87 (22)
- Cancer	0 (0)	10 (1)	0 (0)	1 (2)	8 (2)
Moderate alcohol consumption, n (%)*	25 (93)	278 (41)	7 (88)	24 (41)	NA
**Zutphen Elderly Study**					
N (%)	65 (8)	73 (9)	344 (43)	75 (9)	237 (30)
Age, years	70.5 (5.8)	72.1 (5.0)	72.2 (5.2)	71.8 (5.5)	72.8 (5.3)
Male, n (%)	65 (100)	73 (100)	344 (100)	75 (100)	237 (100)
Alcohol, g/d	12.0 [4.2–24.0]	5.7 [2.1–10.7]	14.0 [5.4–28.0]	18.7 [7.1–41.1]	0 [0–0]
BMI, kg/m^2^	26.0 (3.7)	24.9 (3.7)	25.5 (2.9)	25.3 (2.8)	25.6 (3.1)
Sports, hrs/wk	0.2 (1.0)	0.5 (1.6)	0.4 (1.3)	0.5 (1.6)	0.1 (0.5)
Education, n (%)					
- Low	0 (0)	18 (25)	121 (36)	13 (18)	91 (41)
- Medium	26 (43)	43 (61)	203 (61)	53 (73)	124 (56)
- High	35 (57)	10 (14)	9 (3)	7 (10)	7 (3)
Smoking status, n (%)					
- Never	6 (10)	12 (17)	32 (10)	13 (19)	51 (24)
- Former	36 (58)	41 (59)	162 (49)	37 (53)	101 (47)
- Current	20 (32)	16 (23)	134 (41)	20 (29)	64 (30)
Self-reported medical history, n (%)					
- CHD	5 (8)	13 (18)	60 (17)	9 (12)	53 (22)
- Cancer	3 (5)	7 (10)	24 (7)	7 (9)	25 (11)
Moderate alcohol consumption, n (%)*	33 (51)	57 (78)	147 (43)	28 (37)	NA

Shown are mean (SD), median [Inter-Quartile Range], or n (%)

* Defined as an alcohol consumption >0–12 grams/day.

Most men had a spirit or beer preference; most women had a wine preference or were non-consumers. Furthermore, those with a spirit or no preference had the highest absolute alcohol consumption; except in EPIC-Elderly Spain and SENECA Southern-Europe, where those with a wine preference consumed the highest amount. In the Zutphen Elderly Study, SENECA Northern-Europe, and EPIC-Elderly Sweden, those who preferred wine also had the highest educational attainment. Non-consumers were most likely to have never smoked, whereas those with a spirit preference were most likely to be a current smoker. In all cohorts, age did not differ across preference categories.

### Alcoholic beverage preference and dietary habits: cohort-specific differences

No clinically relevant differences between dietary habits and alcoholic beverage preference were observed in EPIC-Elderly Spain (Table 2). With the only exception that persons with a spirit preference had a higher meat intake than persons with a beer preference. In EPIC-Elderly the Netherlands, also few differences were observed. Persons who preferred beer had a lower dairy intake than persons who preferred wine or non-consumers. Beer consumers also had the lowest intake of mono- and disaccharides, non-consumers the highest. In EPIC-Elderly Greece, persons with a wine preference displayed a higher HDI score than persons with a beer or spirit preference. Furthermore, they had a lower intake of poly-unsaturated fatty acids. Next, persons with a spirit preference had the highest meat intake in EPIC-Elderly Sweden and a lower HDI score compared to persons who preferred wine. In the Rotterdam Study, beer consumers had the highest intake of sugary drinks and saturated fatty acids. Non-consumers had the highest intake of mono- and disaccharides. In SENECA Northern Europe, persons with a wine and spirit preference had a higher fish intake than persons with a beer preference. Non-consumers had a higher intake of mono- and disaccharides compared to persons without a specific preference. Most differences in food and nutrient intakes were observed in SENECA Southern Europe. Here, persons with a spirit preference had higher intakes of fruit, dairy, protein, and mono- and disaccharides, but lower intakes of poly-unsaturated fatty acids. Persons with a wine preference had a higher vegetable intake than persons with a beer preference and a higher oil and fat intake than those with a spirit preference. In the Zutphen Elderly Study, persons who preferred wine had higher fruit and vegetable intake, and a higher adherence to the HDI score than persons who preferred beer. Furthermore, persons with a wine preference and non-consumers consumed more mono- and disaccharides.

**Table 2:** Adjusted ^a^ food group and nutrient intakes (mean (SE)) across categories of alcoholic beverage preference.

	Beer preference	Wine preference	Spirit preference	No preference	Non-consumers
**EPIC-Elderly Spain**					
N (%)	263 (6)	2140 (47)	55 (1)	277 (6)	1857 (40)
Fruit, g/d	338 (14)	325 (5)	349 (30)	332 (14)	351 (6)
Vegetables, g/d	239 (9)	232 (3)	230 (19)	251 (9)	226 (4)
Dairy, g/d	284 (11)	301 (4)	304 (25)	276 (11)	322 (5)
Fish, g/d	54 (2)	57 (1)	65 (5)	57 (2)	57 (1)
Meat, g/d	104 (3)	113 (1)	122 (7)	105 (3)	109 (1)
Oils and fats, g/d	26 (1)	29 (0)	28 (2)	28 (1)	27 (0)
Sugary drinks, g/d	39 (7)	51 (3)	56 (14)	59 (6)	44 (3)
Non-alcohol energy, kcal/d	1944 (6)	1920 (2)	1984 (14)	1940 (6)	1939 (3)
Healthy Diet Indicator, score	46.6 (0.5)	45.2 (0.2)	45.3 (1.1)	45.2 (0.5)	45.4 (0.2)
- Saturated fat, en%	10.8 (0.2)	10.5 (0.1)	10.8 (0.4)	11.1 (0.2)	10.3 (0.1)
- PUFA, en%	5.2 (0.2)	5.6 (0.1)	5.9 (0.3)	5.5 (0.2)	5.4 (0.1)
- Protein, en%	18.9 (0.2)	19.1 (0.1)	19.7 (0.4)	18.9 (0.2)	19.5 (0.1)
- Mono- and disaccharides, en%	18.1 (0.4)	17.5 (0.1)	18.4 (0.8)	18.3 (0.4)	19.2 (0.2)
- Cholesterol, mg/d	331 (7)	344 (3)	351 (14)	340 (7)	336 (3)
- Dietary fiber, g/d	24.5 (0.4)	23.9 (0.2)	24.8 (0.9)	24.4 (0.4)	24.5 (0.2)
- Fruit and vegetables, g/d	577 (18)	556 (7)	579 (39)	583 (17)	577 (8)
**EPIC-Elderly Netherlands**					
N (%)	127 (2)	3783 (58)	541 (8)	708 (11)	1388 (21)
Fruit, g/d	221 (13)	242 (2)	222 (6)	225 (5)	242 (4)
Vegetables, g/d	134 (5)	136 (1)	138 (2)	134 (2)	133 (1)
Dairy, g/d	368 (21)	448 (4)	433 (10)	431 (9)	476 (6)
Fish, g/d	5 (0)	5 (0)	5 (0)	5 (0)	5 (0)
Meat, g/d	88 (4)	84 (1)	88 (2)	88 (2)	83 (1)
Oils and fats, g/d	27 (1)	23 (0)	25 (0)	24 (0)	24 (0)
Sugary drinks, g/d	137 (13)	143 (2)	134 (6)	145 (5)	154 (4)
Non-alcohol energy, kcal/d	1715 (4)	1713 (1)	1715 (2)	1708 (2)	1734 (1)
Healthy Diet Indicator, score	44.7 (0.6)	44.5 (0.1)	44.3 (0.3)	44.2 (0.2)	44.4 (0.2)
- Saturated fat, en%	14.0 (0.2)	13.8 (0.0)	14.1 (0.1)	14.0 (0.1)	13.7 (0.1)
- PUFA, en%	6.4 (0.2)	6.1 (0.0)	6.2 (0.1)	6.1 (0.1)	6.0 (0.0)
- Protein, en%	17.4 (0.3)	17.7 (0.0)	17.6 (0.1)	17.6 (0.1)	17.8 (0.1)
- Mono- and disaccharides, en%	23.7 (0.5)	24.7 (0.1)	23.8 (0.2)	24.0 (0.2)	25.9 (0.2)
- Cholesterol, mg/d	210 (5)	206 91)	212 (3)	211 (2)	202 (2)
- Dietary fiber, g/d	21.6 (0.4)	22.3 (0.1)	22.1 (0.2)	21.6 (0.2)	22.4 (0.1)
- Fruit and vegetables, g/d	355 (14)	377 (3)	360 (7)	359 (6)	375 (4)
**EPIC-Elderly Greece**					
N (%)	537 (6)	2715 (32)	378 (4)	2015 (24)	2773 (33)
Fruit, g/d	342 (6)	351 (3)	319 (8)	335 (3)	336 (3)
Vegetables, g/d	401 (6)	411 (2)	393 (7)	403 (3)	409 (3)
Dairy, g/d	200 (5)	171 (2)	199 (6)	192 (3)	194 (2)
Fish, g/d	15 (1)	19 (0)	15 (1)	18 (0)	18 (0)
Meat, g/d	72 (1)	69 (1)	76 (1)	71 (10	71 (1)
Oils and fats, g/d	47 (0)	47 (0)	47 (1)	47 (0)	48 (0)
Sugary drinks, g/d	103 (4)	90 (2)	100 (5)	95 (2)	95 (2)
Non-alcohol energy, kcal/d	1788 (4)	1779 (2)	1790 (4)	1775 (2)	1796 (2)
Healthy Diet Indicator, score	52.3 (0.3)	54.9 (0.1)	51.2 (0.4)	52.8 (0.2)	53.4 (0.1)
- Saturated fat, en%	11.9 (0.1)	11.5 (0.0)	12.1 (0.1)	11.8 (0.1)	11.8 (0.0)
- PUFA, en%	7.2 (0.1)	5.7 (0.1)	8.6 (0.2)	6.9 (0.1)	6.5 (0.1)
- Protein, en%	15.2 (0.1)	14.9 (0.0)	15.5 (0.1)	15.3 (0.0)	15.3 (0.0)
- Mono- and disaccharides, en%	17.3 (0.2)	17.1 (0.1)	16.7 (0.2)	17.2 (0.1)	17.0 (0.1)
- Cholesterol, mg/d	172 (2)	165 (1)	175 (3)	171 (1)	168 (1)
- Dietary fiber, g/d	20.0 (0.1)	20.6 (0.1)	19.7 (0.2)	19.9 (0.1)	20.4 (0.1)
- Fruit and vegetables, g/d	743 (9)	761 (4)	712 (11)	737 (5)	746 (4)
**EPIC-Elderly Sweden**					
N (%)	963 (30)	416 (13)	139 (4)	1329 (41)	416 (13)
Fruit, g/d	163 (4)	183 (6)	157 (10)	176 (3)	156 (6)
Vegetables, g/d	80 (2)	94 (4)	75 (6)	85 (2)	84 (4)
Dairy, g/d	408 (6)	418 (10)	431 (17)	406 (5)	417 (10)
Fish, g/d	8 (0)	9 (1)	8 (1)	8 (0)	9 (1)
Meat, g/d	63 (1)	63 (2)	70 (3)	67 (1)	61 (2)
Oils and fats, g/d	31 (0)	31 (1)	32 (1)	32 (0)	32 (1)
Sugary drinks, g/d	116 (5)	110 (7)	103 (13)	113 (4)	128 (8)
Non-alcohol energy, kcal/d	1621 (1)	1613 (1)	1620 (2)	1612 (0)	1627 (1)
- Healthy Diet Indicator, score	45.6 (0.2)	45.8 (0.3)	43.8 (0.6)	45.8 (0.2)	44.9 (0.3)
- Saturated fat, en%	14.2 (0.1)	13.9 (0.2)	14.8 (0.3)	14.2 (0.1)	14.4 (0.2)
- PUFA, en%	4.2 (0.0)	4.2 (0.0)	4.2 (0.1)	4.3 (0.0)	4.2 (0.1)
- Protein, en%	14.7 (0.1)	15.0 (0.1)	15.1 (0.2)	14.9 (0.1)	14.6 (0.1)
- Mono- and disaccharides, en%	20.6 (0.2)	21.4 (0.3)	20.7 (0.5)	20.4 (0.1)	21.4 (0.3)
- Cholesterol, mg/d	167 (1)	167 (2)	175 (4)	169 (1)	168 (2)
- Dietary fiber, g/d	19.0 (0.1)	19.5 (0.4)	18.2 (0.4)	19.1 (0.1)	18.9 (0.2)
- Fruit and vegetables, g/d	242 (5)	277 (8)	232 (13)	261 (4)	240 (8)
**Rotterdam Study**					
N (%)	209 (6)	1553 (43)	724 (20)	453 (13)	632 (18)
Fruit, g/d	221 (9)	243 (4)	231 (5)	237 (6)	245 (6)
Vegetables, g/d	213 (7)	215 (3)	212 (4)	213 (5)	203 (4)
Dairy, g/d	383 (18)	401 (7)	392 (0)	373 (12)	406 (10)
Fish, g/d	17 (1)	16 (0)	16 (1)	16 (1)	15 (1)
Meat, g/d	113 (3)	109 (1)	110 (2)	106 (2)	103 (2)
Oils and fats, g/d	41 (1)	40 (0)	40 (1)	41 (1)	39 (1)
Sugary drinks, g/d	241 (17)	131 (6)	120 (10)	136 (11)	143 (10)
Non-alcohol energy, kcal/d	1918 (4)	1898 (2)	1882 (3)	1892 (3)	1924 (3)
Healthy Diet Indicator, score	43.5 (0.4)	43.7 (0.2)	44.0 (0.3)	44.5 (0.3)	43.9 (0.3)
- Saturated fat, en%	14.5 (0.2)	14.3 (0.1)	14.3 (0.1)	14.4 (0.1)	13.9 (0.1)
- PUFA, en%	6.8 (0.2)	6.7 (0.1)	7.1 (0.1)	7.2 (0.1)	6.7 (0.1)
- Protein, en%	17.1 (0.2)	16.9 (0.1)	16.9 (0.1)	16.7 (0.1)	16.7 (0.1)
- Mono- and disaccharides, en%	21.3 (0.4)	21.8 (0.2)	20.8 (0.2)	21.1 (0.3)	23.5 (0.2)
- Cholesterol, mg/d	235 (4)	236 (2)	231 (3)	228 (3)	227 (3)
- Dietary fiber, g/d	16.3 (0.3)	17.1 (0.1)	17.0 (0.2)	17.4 (0.2)	16.9 (0.2)
- Fruit and vegetables, g/d	434 (13)	458 (5)	442 (7)	450 (9)	447 (7)
**SENECA: Northern Europe**					
N (%)	140 (13)	312 (29)	104 (10)	112 (10)	401 (38)
Fruit, g/d	183 (12)	177 (9)	195 (14)	181 (14)	202 (7)
Vegetables, g/d	302 (10)	293 (7)	266 (12)	281 (12)	310 (6)
Dairy, g/d	293 (19)	290 (13)	305 (22)	309 (21)	338 (12)
Fish, g/d	16 (2)	24 (1)	26 (2)	23 (2)	21 (1)
Meat, g/d	133 (5)	126 (3)	125 (5)	128 (5)	121 (3)
Oils and fats, g/d	46 (2)	45 (1)	46 (2)	45 (2)	44 (1)
Non-alcohol energy, kcal/d	2039 (49)	2015 (34)	2021 (56)	2062 (54)	2043 (29)
Healthy Diet Indicator, score	46.3 (0.6)	46.8 (0.4)	45.8 (0.7)	47.1 (0.7)	46.6 (0.4)
- Saturated fat, en%	16.9 (0.3)	16.1 (0.2)	16.8 (0.4)	17.0 (0.4)	16.2 (0.2)
- PUFA, en%	6.2 (0.3)	6.6 (0.2)	7.0 (0.3)	6.9 (0.3)	6.8 (0.2)
- Protein, en%	13.7 (0.2)	14.0 (0.2)	14.0 (0.3)	14.0 (0.2)	14.2 (0.1)
- Mono- and disaccharides, en%	19.4 (0.5)	18.9 (0.4)	19.5 (0.6)	18.0 (0.6)	20.4 (0.3)
- Cholesterol, mg/d	313 (9)	315 (6)	311 (10)	312 (10)	304 (6)
- Dietary fiber, g/d	22.0 (0.6)	22.2 (0.4)	21.7 (0.6)	21.6 (0.6)	23.5 (0.3)
- Fruit and vegetables, g/d	486 (16)	471 (11)	460 (19)	462 (18)	512 (10)
**SENECA: Southern Europe**					
N (%)	27 (2)	682 (58)	8 (1)	58 (5)	394 (34)
Fruit, g/d	274 (39)	303 (9)	371 (72)	250 (27)	291 (12)
Vegetables, g/d	223 (28)	278 (6)	270 (52)	246 (20)	259 (9)
Dairy, g/d	283 (40)	266 (9)	348 (72)	271 (27)	288 (13)
Fish, g/d	41 (7)	46 (2)	42 (13)	44 (5)	37 (2)
Meat, g/d	126 (10)	104 (2)	92 (18)	115 (7)	104 (3)
Oils and fats, g/d	28 (4)	32 (1)	20 (7)	34 (3)	33 (1)
Non-alcohol energy, kcal/d	1980 (114)	2009 (25)	1638 (208)	1992 (79)	1882 (36)
Healthy Diet Indicator, score	46.3 (1.6)	48.6 (0.3)	46.9 (2.9)	46.4 (1.1)	47.8 (0.5)
- Saturated fat, en%	12.0 (0.6)	11.4 (0.1)	11.7 (1.1)	11.8 (0.4)	11.6 (0.2)
- PUFA, en%	4.7 (0.5)	5.3 (0.1)	3.7 (1.0)	5.8 (0.4)	5.1 (0.2)
- Protein, en%	15.7 (0.5)	15.4 (0.1)	16.7 (1.0)	15.3 (0.4)	15.5 (0.2)
- Mono- and disaccharides, en%	19.4 (1.4)	16.3 (0.3)	22.6 (2.5)	17.6 (0.9)	17.6 (0.4)
- Cholesterol, mg/d	305 (20)	278 (4)	275 (37)	305 (14)	292 (6)
- Dietary fiber, g/d	19.2 (1.7)	20.2 (0.4)	20.0 (3.0)	19.1 (1.1)	19.1 (0.5)
- Fruit and vegetables, g/d	496 (52)	581 (11)	640 (95)	496 (36)	551 (16)
**Zutphen Elderly Study**					
N (%)	65 (9)	73 (9)	344 (43)	75 (9)	237 (30)
Fruit, g/d	170 (17)	242 (16)	193 (8)	194 (16)	200 (10)
Vegetables, g/d	171 (9)	174 (9)	179 (4)	168 (9)	173 (5)
Dairy, g/d	362 (29)	364 (27)	385 (13)	324 (27)	394 (17)
Fish, g/d	16 (3)	19 (3)	22 (1)	19 (3)	15 (2)
Meat, g/d	119 (5)	113 (5)	114 (2)	116 (5)	108 (3)
Oils and fats, g/d	50 (2)	45 (2)	50 (1)	50 (2)	51 (1)
Sugary drinks, g/d	60 (12)	68 (11)	56 (6)	52 (11)	61 (7)
Non-alcohol energy, kcal/d	2271 (63)	2199 (60)	2135 (29)	2181 (60)	2138 (37)
Healthy Diet Indicator, score	42.5 (0.8)	45.6 (0.8)	44.6 (0.4)	44.4 (0.8)	44.4 (0.5)
- Saturated fat, en%	17.4 (0.4)	16.8 (0.4)	17.1 (0.2)	17.5 (0.4)	16.7 (0.3)
- PUFA, en%	6.9 (0.3)	6.5 (0.3)	7.0 (0.2)	6.7 (0.3)	7.1 (0.2)
- Protein, en%	14.8 (0.3)	14.6 (0.3)	15.0 (0.1)	14.2 (0.3)	14.5 (0.2)
- Mono- and disaccharides, en%	20.6 (0.8)	22.5 (0.7)	20.5 (0.3)	20.1 (0.7)	22.5 (0.4)
- Cholesterol, mg/d	306 (11)	286 (10)	290 (5)	299 (10)	276 (6)
- Dietary fiber, g/d	24.3 (0.8)	25.5 (0.7)	24.8 (0.3)	23.5 (0.7)	24.5 (0.4)
- Fruit and vegetables, g/d	341 (20)	416 (19)	372 (9)	362 (19)	373 (12)

Abbreviations: poly-unsaturated fatty acids (PUFA)

^a^ Adjusted for age, sex, education, employment, self-reported prevalence of CHD or cancer, energy intake, smoking status, moderate alcohol consumption, and physical activity.

### Alcoholic beverage preference and dietary habits: overall comparison

Only few relevant differences in food and nutrient intake were observed in the separate cohorts. Still some general trends were observed. Persons with a wine preference had a higher HDI score in EPIC-Elderly Greece, the Zutphen Elderly Study, and EPIC-Elderly Sweden and a higher intake of fish, vegetables, dairy, oils and fats, and fruit in some cohorts. In the Dutch cohorts and SENECA Northern Europe, non-consumers had the highest intake of mono- and disaccharides. Next, persons with a spirit preference had a higher meat consumption in EPIC-Elderly Spain and Sweden. Persons who preferred beer had the highest intake of sugary drinks and saturated fatty acids in the Rotterdam Study, a higher meat intake in SENECA Southern Europe, but lowest intake of mono- and disaccharides in EPIC-Elderly the Netherlands.

Combined weighted estimates for all cohorts combined are displayed in Table 3. Persons with a wine preference constituted the largest group. The overall comparison between preference category and diet showed somewhat different results than the cohort-specific comparisons. Overall, non-consumers had the highest fruit and vegetable intake, followed by wine consumers. Persons with a beer preference had the lowest intake of vegetables, PUFA, and protein. Persons who preferred spirits had highest intake of meat and spirits. Although variation between the categories was small, persons with no specific preference and non-consumers had the highest HDI score; spirit consumers the lowest.

**Table 3:** Adjusted ^a^ food group and nutrient intakes (weighted mean and SE) across categories of alcoholic beverage preference of all cohorts combined.

	Beer preference	Wine preference	Spirit preference	No preference	Non-consumers
All (n = 29,423)^a^					
N (%)	2331 (8)	11674 (40)	2293 (8)	5027 (17)	8098 (28)
Fruit, g/d	214 (7)	272 (4)	230 (8)	247 (5)	276 (5)
Vegetables, g/d	148 (4)	171 (2)	170 (4)	172 (3)	194 (3)
Dairy, g/d	289 (8)	267 (4)	307 (10)	253 (5)	260 (4)
Fish, g/d	10 (1)	7 (0)	7 (0)	9 (0)	9 (0)
Meat, g/d	75 (2)	82 (1)	91 (2)	75 (1)	80 (1)
Oils and fats, g/d	36 (1)	33 (0)	36 (1)	38 (0)	37 (0)
Sugary drinks, g/d	99 (6)	96 (2)	95 (6)	100 (3)	91 (3)
Non-alcohol energy, kcal/d	1639 (1)	1721 (1)	1735 (2)	1638 (1)	1728 (1)
Healthy Diet Indicator, score	46.9 (0.4)	47.3 (0.2)	45.4 (0.4)	48.1 (0.2)	48.0 (0.2)
- Saturated fat, en%	13.2 (0.1)	12.6 (0.1)	13.9 (0.2)	12.8 (0.1)	12.4 (0.1)
- PUFA, en%	4.5 (0.1)	5.7 (0.1)	6.0 (0.1)	4.9 (0.1)	5.5 (0.1)
- Protein, en%	15.3 (0.1)	16.2 (0.1)	16.1 (0.1)	15.5 (0.1)	16.0 (0.1)
- Mono- and disaccharides, en%	19.3 (0.2)	20.1 (0.1)	20.1 (0.3)	19.0 (0.2)	19.5 (0.2)
- Cholesterol, mg/d	183 (2)	203 (1)	213 (3)	183 (2)	197 (2)
- Dietary fiber, g/d	19.7 (0.2)	20.9 (0.1)	19.9 (0.2)	19.9 (0.1)	20.9 (0.1)
- Fruit and vegetables, g/d	380 (9)	477 (4)	422 (9)	447 (6)	513 (6)

^a^ Adjusted for age, sex, education, employment, self-reported prevalence of CHD or cancer, energy intake, smoking status, moderate alcohol consumption, and physical activity.

### Sensitivity analyses

Persons with self-reported prevalent diabetes were excluded because their diagnosis may have affected their alcohol consumption and dietary habits. However, including persons with diabetes in the analyses did not influence the results (S1 Table). Excluding all persons with self-reported prevalent CHD, diabetes, or cancer at baseline did also not affect the estimates. Men who preferred wine had the highest HDI score, including lower saturated fat intake and higher fruit and vegetable intake, whereas in women, non-consumers displayed the highest HDI scores, as well as the lowest saturated fat intake and highest fruit and vegetable intake.

Overweight and obese persons had a higher HDI score and higher absolute intakes of food groups, but dietary habits according to preference category were similar. Persons with a lower alcohol consumption had higher HDI scores and intakes of most components; persons with a higher alcohol consumption had higher absolute intakes of most food groups. However, the differences in dietary intake and diet quality between preference categories remained similar.

## Discussion

In this study among 29,423 elderly participants from 14 European countries, dietary habits and diet quality did not differ greatly according to alcoholic beverage preference. Persons with a wine preference tended to have a higher diet quality and intake of healthy foods in some cohorts. Combining all cohorts, non-consumers and persons without a specific preference had highest diet quality. Overall, this large study of harmonized alcohol and dietary data from different European countries showed no consistent dietary patterns associated with alcoholic beverage preference after adjustment for socio-demographic and lifestyle factors.

These findings give insight in the associations between the alcoholic beverage preference and diet and the potential confounding effect of diet, which may be used in future studies into beverage-specific health effects. Light to moderate alcohol intake has been suggested to have a beneficial effect, especially on CHD [28]. Furthermore, studies tend to find a larger protective effect of wine consumption compared to other specific beverages [29]. The mechanisms by which moderate alcohol consumption decreases coronary artery disease include the increase of high density lipoprotein levels, the modification of hemostatic parameters, and the inhibition of lipoprotein oxidation by phenolic components present in red wine. Although a biological effect of alcohol exists, differences in consumption patterns and lifestyle can also partly account for the protective effect of a moderate alcohol consumption. Differential effects of beer, wine, and spirits could be more likely to reflect underlying differences in socio-demographic factors and lifestyle of the study population [30]. Furthermore, it is also likely that health effects are at least partly driven by the beverage that is consumed most in the country or population under study. Thus, the association between alcohol consumption and health outcomes is likely to be confounded by socio-demographic and lifestyle factors. Indeed, most scientific evidence on the relationship between alcohol consumption and health outcomes including CVD and mortality is based upon observational cohort studies where confounding by age, sex, socioeconomic status and lifestyle is present. Of the 18 prospective studies included in the meta-analysis by Constanzo *et al*. on the association between beer, wine, spirits, and non-fatal cardiovascular events, three of them adjusted for age only. The other 15 studies included a wide range of covariates, including age, sex, education, smoking, BMI, and prevalent diseases, but only three studies included adjustment for dietary intake. The exact impact of adjustment for dietary factors could not be determined from these studies [1]. Therefore, this study focused on whether dietary habits differed between persons with a preference for beer, wine, or spirits, and thus the potential confounding effect of diet in the relationship between alcohol and health outcomes.

Several studies have investigated the associations between alcoholic beverage preference and dietary habits across different countries. In a large Danish sample. Tjønneland *et al*. observed that wine consumption was strongly associated with a healthier diet [5]. In a study among 3,756 American men and women, beer and liquor drinkers had a lower intake of fruit, vegetables and grains and wine consumers had a higher intake of fruit, juices, and vegetables, also after adjustment for age, education, and smoking status [10]. In another study among American subjects, wine drinkers had healthier diets, characterized by a higher fruit and vegetable intake and a lower intake of meat [31]. However, a number of studies also found less distinct associations. Although wine drinkers reported higher intake of fibre and olive oil and lower intake of sugary drinks and fast foods compared to other alcoholic beverage groups, no relevant differences in adherence to the Mediterranean food pattern was observed among 10,526 Spanish university graduates [32]. Chatenoud *et al*. did not observe a relationship between wine drinking and indicators of a healthy diet, including intake of fruit, vegetables, and fish, in a sample of Italian adults [33]. In the study of Männistö *et al*. among Finnish men and women, alcoholic beverage preference was not associated with macronutrient intakes, except that women who preferred wine consumed more carotenoids [34]. The study of Carmona-Torre *et al*. among Mediterranean adults showed no substantial differences in adherence to the Mediterranean diet and type of alcoholic beverage consumed [35]. In conclusion, previous studies have used different definitions of alcoholic beverage preference and a wide range of dietary exposures. Nevertheless, largest differences were found between persons who preferred wine and those who preferred beer. In studies performed in both Western and Mediterranean populations, persons with a beer preference displayed unhealthier dietary habits. Persons with a wine preference had a healthier diet in Western populations, but this was generally not observed in Mediterranean countries [6].

The Healthy Diet Indicator was included because diet quality gives more insight in the potential confounding effect of diet regarding the association between alcohol and health outcomes. In a study by Jankovic *et al*., performed earlier within 11 cohort studies from the CHANCES project, a 10-point increase in HDI score was associated with a reduced mortality risk of HR 0.90 (95%CI 0.87, 0.93) in 396,391 participants. One way to evaluate the validity of the HDI score is to assess the relationship with health outcomes [36]. It was therefore concluded that the HDI score as a measure of adherence to the WHO dietary guidelines is a useful tool in international comparison studies [20]. No large differences in HDI scores and alcoholic beverage preference were observed within the CHANCES cohorts. In contrast, other studies found that wine consumption was associated with a higher diet quality. A recent study among 1,699 Belgian military men showed that beer consumers had lower scores of the Healthy Eating Index (HEI) 2010 compared to wine consumers [37]. Forshee *et al*. also showed that wine consumption was positively associated with the HEI, although no adjustments were performed [38]. Moreover, Ruidavets *et al*. investigated a diet quality index based upon French nutritional recommendations and beverage preference among 1,100 middle-aged men. Diet quality was higher among wine drinkers, compared to beer and mixed drinkers after multivariate adjustment [25].

The comparison with previous studies has shown that associations between alcoholic beverage preference and dietary habits vary across countries and cultures. Specifically, the dietary habits related to wine preference differ between Western and Mediterranean populations. As a result, the strength and direction of diet’s potential as a confounder may be highly influenced by the cultural norms in the study population. For example, within EPIC-Elderly Spain persons with a beer preference tended to have higher HDI scores, whereas most other cohorts showed a higher HDI score for persons with a wine preference. Although the choice of alcoholic beverage is determined by cultural norms, these differences are also explained by socioeconomic status. In wine producing countries such as Spain, wine is affordable for all socioeconomic groups, and part of the traditional Mediterranean diet. On the other hand, in Northern European countries, wine is relatively expensive and thus affordable for individuals with a higher social status. As a result, socioeconomic status might be an important factor in the apparent health effects of wine [39]. Indeed, after adjustment for educational attainment and employment status next to the other confounders no large differences in dietary habits were seen across preference categories.

A strength of the CHANCES project is that it is a large-scale multi-national collaboration of cohort studies. Pooled analyses of the individual participant data from these different cohorts is a cost-efficient analytical approach and increases statistical power considerably. However, although a large effort has been made to harmonize the data across the cohorts, the study relies on secondary data collected according to different study objectives and protocols, which may be considered a weakness [13].

The inconsistent results of the current as well as previous studies may indicate that (residual) confounding by in particular socio-economic status hampers the findings. Alcoholic beverage preference, diet, but also smoking behavior, physical activity are determined by age, sex, socio-economic status and cultural or religious norms. Hence, when the specific health effects of wine, beer, and spirits are of interest, these socio-demographic determinants act as confounders. If these factors are accurately measured and adequately controlled for in the analysis, the association between alcoholic beverage preference and health outcomes are probably not biased by diet. Most studies also corrected for age, sex, educational status, and lifestyle factors. However, confounding factors can only be fully accounted for if they are measured accurately, which is usually unlikely to be true. To provide a reliable and correct measure of dietary intake is particularly challenging in epidemiological studies [40]. As a result, observational studies, including the present one, may never be able to fully adjust for the confounding factors associated with alcoholic beverage consumption. Furthermore, it is difficult to disentangle the effects of volume, frequency and preference of alcoholic beverage intake because of their correlation. Therefore, genetic and metabolic studies might be more suited than observational studies to provide further evidence on the causal link between consumption of alcoholic beverages and disease risk.

In conclusion, this study using harmonized data from ~30,000 elderly from 14 European countries showed that dietary intake and diet quality did not differ greatly according to alcoholic beverage preference after multivariate adjustment. Nevertheless, since alcohol is a complex exposure, future studies into alcohol consumption and health effects should carefully consider all associated socio-economic and lifestyle factors, including diet.

## Supporting Information

S1 Table Adjusted food group and nutrient intakes (weighted mean and SE) across categories of alcoholic beverage preference in those excluding prevalent diseases, and stratified by gender, BMI-category, and alcohol consumption category. (DOCX)
