# Supplementary material for: Alcoholic Beverage Preference and Dietary Habits in Elderly across Europe: Analyses within the Consortium on Health and Ageing: Network of Cohorts in Europe and the United States (CHANCES) Project
Source: PLoS One. 2016 Aug 22;11(8):e0161603. doi: 10.1371/journal.pone.0161603 (PMC4993358; doi:10.1371/journal.pone.0161603)
Supplement: S1 Table — (DOCX) [file pone.0161603.s001.docx]

**Supporting information**

**S1 Table.** **Adjusted food group and nutrient intakes (weighted mean and SE) across categories of alcoholic beverage preference in those excluding prevalent diseases, and stratified by gender, BMI-category, and alcohol consumption category.**

|  | Beer preference | Wine preference | Spirit preference | No preference | Non-consumers |
| --- | --- | --- | --- | --- | --- |
| Excluding prevalent diseases (n=26,121) ^a^ |  |  |  |  |  |
| N (%) | 2070 (8) | 10514 (40) | 1889 (7) | 4557 (17) | 7091 (27) |
| Fruit, g/d | 214 (7) | 272 (4) | 230 (8) | 248 (5) | 281 (5) |
| Vegetables, g/d | 147 (4) | 172 (2) | 170 (4) | 173 (3) | 196 (3) |
| Dairy, g/d | 289 (8) | 265 (4) | 305 (110 | 251 (5) | 257 (4) |
| Fish, g/d | 10 (1) | 7 (0) | 7 (0) | 9 (0) | 8 (0) |
| Meat, g/d | 74 (2) | 82 (1) | 91 (2) | 75 (1) | 79 (1) |
| Oils and fats, g/d | 37 (1) | 33 (0) | 36 (1) | 38 (0) | 37 (0) |
| Sugary drinks, g/d | 100 (6) | 96 (3) | 95 (7) | 101 (3) | 90 (3) |
| Non-alcohol energy, kcal/d | 1643 (1) | 1724 (1) | 1731 (3) | 1643 (1) | 1728 (2) |
|  |  |  |  |  |  |
| Healthy Diet Indicator, score | 46.9 (0.4) | 47.2 (0.2) | 45.4 (0.4) | 48.1 (0.2) | 48.1 (0.2) |
| - Saturated fat, en% | 13.2 (0.2) | 12.6 (0.1) | 13.9 (0.2) | 12.8 (0.1) | 12.3 (0.1) |
| - PUFA, en% | 4.5 (0.1) | 5.7 (0.1) | 5.9 (0.1) | 4.9 (0.1) | 5.5 (0.1) |
| - Protein, en% | 15.3 (0.1) | 16.2 (0.1) | 16.1 (0.1) | 15.5 (0.1) | 16.0 (0.1) |
| - Mono- and disaccharides, en% | 19.3 (0.3) | 20.0 (0.1) | 20.1 (0.3) | 18.9 (0.2) | 19.4 (0.2) |
| - Cholesterol, mg/d | 183 (2) | 204 (1) | 213 () | 183 (2) | 197 (2) |
| - Dietary fiber, g/d | 19.8 (0.2) | 21.0 (0.1) | 20.1 (0.3) | 19.9 (0.1) | 20.9 (0.1) |
| - Fruit and vegetables, g/d | 378 (9) | 478 (5) | 421 (10) | 449 (6) | 519 (6) |
|  |  |  |  |  |  |
| Men (n=10,467) ^b^ |  |  |  |  |  |
| N (%) | 1323 (13) | 3597 (34) | 1430 (14) | 2583 (25) | 1534 (15) |
| Fruit, g/d | 181 (8) | 309 (8) | 214 (9) | 223 (6) | 255 (12) |
| Vegetables, g/d | 122 (5) | 275 (6) | 186 (5) | 161 (4) | 218 (7) |
| Dairy, g/d | 319 (12) | 219 (6) | 304 (12) | 255 (7) | 259 (10) |
| Fish, g/d | 13 (1) | 22 (1) | 15 (1) | 16 (1) | 19 (1) |
| Meat, g/d | 91 (2) | 96 (2) | 106 (3) | 89 (2) | 97 (3) |
| Oils and fats, g/d | 44 (1) | 44 (1) | 47 (1) | 47 (1) | 46 (1) |
| Sugary drinks, g/d | 115 (9) | 85 (4) | 92 (8) | 109 (5) | 92 (7) |
| Non-alcohol energy, kcal/d | 1907 (1) | 2029 (4) | 1990 (4) | 1899 (1) | 2005 (5) |
|  |  |  |  |  |  |
| Healthy Diet Indicator, score | 46.2 (0.5) | 49.7 (0.3) | 45.6 (0.5) | 48.1 (0.3) | 47.5 (0.5) |
| - Saturated fat, en% | 13.5 (0.2) | 11.2 (0.1) | 13.8 (0.2) | 12.7 (0.1) | 12.5 (0.2) |
| - PUFA, en% | 4.7 (0.1) | 5.5 (0.1) | 6.2 (0.2) | 5.0 (0.1) | 5.7 (0.2) |
| - Protein, en% | 14.9 (0.1) | 15.7 (0.1) | 15.5 (0.2) | 15.1 (0.1) | 15.5 (0.1) |
| - Mono- and disaccharides, en% | 18.8 (0.4) | 16.4 (0.2) | 18.7 (0.4) | 17.6 (0.2) | 18.0 (0.3) |
| - Cholesterol, mg/d | 218 (4) | 242 (3) | 245 (5) | 216 (3) | 240 (5) |
| - Dietary fiber, g/d | 20.9 (0.3) | 23.2 (0.2) | 20.8 (0.3) | 21.3 (0.2) | 22.4 (0.3) |
| - Fruit and vegetables, g/d | 323 (10) | 613 (10) | 416 (11) | 414 (8) | 502 (15) |
|  |  |  |  |  |  |
| Women (n=18,956) ^b^ |  |  |  |  |  |
| N (%) | 1008 (5) | 8077 (43) | 863 (5) | 2444 (13) | 6564 (35) |
| Fruit, g/d | 242 (10) | 264 (4) | 235 (10) | 260 (6) | 287 (5) |
| Vegetables, g/d | 165 (6) | 158 (1) | 153 (4) | 168 (3) | 193 (2) |
| Dairy, g/d | 264 (10) | 295 (5) | 321 (15) | 251 (6) | 260 (4) |
| Fish, g/d | 8 (1) | 6 (0) | 5 (0) | 7 (0) | 8 (0) |
| Meat, g/d | 62 (2) | 75 (1) | 82 (3) | 65 (1) | 70 (1) |
| Oils and fats, g/d | 30 (1) | 29 (0) | 29 (1) | 32 (0) | 33 (0) |
| Sugary drinks, g/d | 88 (6) | 99 (3) | 108 (9) | 92 (4) | 82 (2) |
| Non-alcohol energy, kcal/d | 1464 (1) | 1605 (1) | 1613 (3) | 1487 (1) | 1633 (1) |
|  |  |  |  |  |  |
| Healthy Diet Indicator, score | 47.7 (0.5) | 46.2 (0.2) | 45.1 (0.5) | 48.1 (0.3) | 48.2 (0.2) |
| - Saturated fat, en% | 12.8 (0.2) | 13.1 (0.1) | 13.8 (0.2) | 12.9 (0.1) | 12.4 (0.1) |
| - PUFA, en% | 4.4 (0.1) | 5.7 (0.0) | 6.0 (0.1) | 4.8 (0.1) | 5.4 (0.1) |
| - Protein, en% | 15.6 (0.1) | 16.6 (0.1) | 16.8 (0.2) | 15.8 (0.1) | 16.2 (0.1) |
| - Mono- and disaccharides, en% | 19.7 (0.3) | 21.6 (0.1) | 21.5 (0.4) | 19.8 (0.2) | 20.2 (0.1) |
| - Cholesterol, mg/d | 153 (3) | 188 (1) | 196 (4) | 159 (2) | 174 (2) |
| - Dietary fiber, g/d | 18.8 (0.3) | 19.9 (0.1) | 19.7 (0.3) | 19.1 (0.2) | 19.7 (0.1) |
| - Fruit and vegetables, g/d | 423 (13) | 447 (4) | 408 (12) | 458 (8) | 523 (6) |
|  |  |  |  |  |  |
| BMI < 25 kg/m^2^ (n=9,149) ^c^ |  |  |  |  |  |
| N (%) | 852 (9) | 3826 (42) | 782 (9) | 1619 (18) | 2070 (23) |
| Fruit, g/d | 188 (10) | 250 (6) | 208 (12) | 219 (8) | 243 (9) |
| Vegetables, g/d | 121 (6) | 151 (2) | 154 (6) | 134 (4) | 159 (4) |
| Dairy, g/d | 333 (15) | 320 (8) | 341 (20) | 299 (10) | 298 (11) |
| Fish, g/d | 8 (1) | 6 (0) | 7 (1) | 7 (0) | 7 (0) |
| Meat, g/d | 69 (2) | 78 (2) | 88 (3) | 70 (2) | 77 (2) |
| Oils and fats, g/d | 34 (1) | 30 (0) | 35 (1) | 34 (1) | 34 (1) |
| Sugary drinks, g/d | 95 (10) | 103 (5) | 87 (10) | 102 (7) | 101 (7) |
| Non-alcohol energy, kcal/d | 1641 (1) | 1720 (2) | 1752 (4) | 1638 (1) | 1731 (2) |
|  |  |  |  |  |  |
| Healthy Diet Indicator, score | 46.1 (0.6) | 46.4 (0.3) | 45.0 (0.6) | 46.9 (0.4) | 46.9 (0.4) |
| - Saturated fat, en% | 13.6 (0.3) | 13.1 (0.1) | 14.4 (0.3) | 13.4 (0.2) | 12.9 (0.2) |
| - PUFA, en% | 4.4 (0.1) | 5.7 (0.1) | 5.9 (0.2) | 4.7 (0.1) | 5.4 (0.1) |
| - Protein, en% | 15.0 (0.2) | 16.1 (0.1) | 15.5 (0.2) | 15.2 (0.1) | 15.6 (0.1) |
| - Mono- and disaccharides, en% | 20.4 (0.4) | 22.1 (0.2) | 21.3 (0.5) | 20.3 (0.3) | 21.7 (0.3) |
| - Cholesterol, mg/d | 177 (3) | 201 (2) | 213 (6) | 180 (3) | 194 (4) |
| - Dietary fiber, g/d | 19.5 (0.4) | 21.1 (0.2) | 19.9 (0.4) | 19.8 (0.3) | 20.9 (0.3) |
| - Fruit and vegetables, g/d | 320 (12) | 422 (6) | 378 (15) | 371 (10) | 434 (11) |
|  |  |  |  |  |  |
| BMI ≥25 kg/m^2^ (n=20,274) ^c^ |  |  |  |  |  |
| N (%) | 1479 (7) | 7848 (39) | 1511 (7) | 3408 (17) | 6028 (30) |
| Fruit, g/d | 234 (9) | 285 (4) | 241 (9) | 262 (6) | 291 (5) |
| Vegetables, g/d | 174 (6) | 187 (2) | 179 (5) | 199 (4) | 214 (3) |
| Dairy, g/d | 270 (9) | 247 (4) | 293 (12) | 238 (5) | 250 (4) |
| Fish, g/d | 11 (1) | 8 (0) | 7 (1) | 10 (0) | 10 (0) |
| Meat, g/d | 78 (2) | 83 (1) | 92 (2) | 77 (1) | 80 (1) |
| Oils and fats, g/d | 38 (1) | 35 (0) | 36 (1) | 40 (0) | 38 (0) |
| Sugary drinks, g/d | 101 (6) | 93 (3) | 99 (8) | 99 (4) | 88 (3) |
| Non-alcohol energy, kcal/d | 1640 (1) | 1726 (2) | 1727 (3) | 1640 (1) | 1733 (2) |
|  |  |  |  |  |  |
| Healthy Diet Indicator, score | 47.4 (0.4) | 47.7 (0.2) | 45.6 (0.4) | 48.6 (0.3) | 48.3 (0.2) |
| - Saturated fat, en% | 12.9 (0.2) | 12.4 (0.1) | 13.7 (0.2) | 12.6 (0.1) | 12.2 (0.1) |
| - PUFA, en% | 4.7 (0.1) | 5.7 (0.1) | 6.1 (0.1) | 5.0 (0.1) | 5.6 (0.1) |
| - Protein, en% | 15.5 (0.1) | 16.3 (0.1) | 16.3 (0.2) | 15.6 (0.1) | 16.2 (0.1) |
| - Mono- and disaccharides, en% | 18.8 (0.3) | 19.2 (0.1) | 19.6 (0.3) | 18.4 (0.2) | 18.8 (0.2) |
| - Cholesterol, mg/d | 187 (3) | 204 (2) | 213 (4) | 184 (2) | 197 (2) |
| - Dietary fiber, g/d | 19.9 (0.3) | 20.8 (0.1) | 19.8 (0.3) | 19.9 (0.1) | 20.8 (0.1) |
| - Fruit and vegetables, g/d | 427 (12) | 513 (6) | 447 (12) | 494 (8) | 550 (7) |
|  |  |  |  |  |  |
| Alcohol consumption <0-12 g/day (n=22,561) ^d^ |  |  |  |  |  |
| N (%) | 1936 (13) | 7723 (53) | 1103 (8) | 3701 (26) | NA |
| Fruit, g/d | 214 (7) | 269 (4) | 226 (11) | 242 (5) | NA |
| Vegetables, g/d | 139 (4) | 166 (2) | 157 (5) | 161 (3) | NA |
| Dairy, g/d | 294 (8) | 278 (4) | 312 (14) | 259 (5) | NA |
| Fish, g/d | 10 (1) | 7 (0) | 7 (1) | 8 (0) | NA |
| Meat, g/d | 69 (2) | 77 (1) | 85 (3) | 70 (1) | NA |
| Oils and fats, g/d | 35 (1) | 33 (0) | 35 (1) | 37 (0) | NA |
| Sugary drinks, g/d | 98 (6) | 97 (3) | 85 (9) | 97 (4) | NA |
| Non-alcohol energy, kcal/d | 1691 (1) | 2018 (1) | 1760 (2) | 1686 (1) | NA |
|  |  |  |  |  |  |
| Healthy Diet Indicator, score | 47.2 (0.4) | 47.2 (0.2) | 45.7 (0.5) | 48.3 (0.3) | NA |
| - Saturated fat, en% | 13.2 (0.2) | 13.0 (0.1) | 14.1 (0.2) | 13.1 (0.1) | NA |
| - PUFA, en% | 4.5 (0.1) | 5.7 (0.1) | 5.6 (0.1) | 4.7 (0.0) | NA |
| - Protein, en% | 15.4 (0.1) | 16.3 (0.1) | 16.2 (0.2) | 15.6 (0.1) | NA |
| - Mono- and disaccharides, en% | 19.7 (0.3) | 21.0 (0.1) | 20.6 (0.4) | 19.4 (0.2) | NA |
| - Cholesterol, mg/d | 174 (2) | 193 (2) | 202 (4) | 174 (2) | NA |
| - Dietary fiber, g/d | 19.5 (0.2) | 20.4 (0.1) | 19.9 (0.3) | 19.7 (0.1) | NA |
| - Fruit and vegetables, g/d | 370 (9) | 468 (5) | 405 (13) | 430 (6) | NA |
|  |  |  |  |  |  |
| Alcohol consumption ≥12 g/day (n=6562) ^d^ |  |  |  |  |  |
| N (%) | 395 (6) | 3951 (60) | 1190 (18) | 1026 (16) | NA |
| Fruit, g/d | 210 (19) | 279 (6) | 224 (9) | 258 (10) | NA |
| Vegetables, g/d | 200 (12) | 193 (3) | 193 (5) | 215 (6) | NA |
| Dairy, g/d | 262 (23) | 237 (6) | 295 (12) | 232 (9) | NA |
| Fish, g/d | 11 (1) | 9 (0) | 9 (1) | 11 (1) | NA |
| Meat, g/d | 109 (5) | 105 (2) | 109 (3) | 98 (3) | NA |
| Oils and fats, g/d | 41 (2) | 35 (0) | 39 (1) | 42 (1) | NA |
| Sugary drinks, g/d | 101 (16) | 86 (4) | 113 (9) | 111 (7) | NA |
| Non-alcohol energy, kcal/d | 1960 (14) | 1909 (5) | 1911 (7) | 1913 (8) | NA |
|  |  |  |  |  |  |
| Healthy Diet Indicator, score | 45.4 (0.9) | 47.0 (0.3) | 44.6 (0.4) | 47.4 (0.5) | NA |
| - Saturated fat, en% | 12.8 (0.4) | 11.2 (0.1) | 14.4 (0.2) | 11.9 (0.2) | NA |
| - PUFA, en% | 5.5 (0.3) | 5.6 (0.1) | 6.5 (0.2) | 5.6 (0.1) | NA |
| - Protein, en% | 15.1 (0.3) | 15.9 (0.1) | 15.4 (0.2) | 14.9 (0.1) | NA |
| - Mono- and disaccharides, en% | 17.7 (0.7) | 16.8 (0.2) | 18.3 (0.3) | 16.9 (0.3) | NA |
| - Cholesterol, mg/d | 247 (9) | 253 (3) | 242 (4) | 231 (5) | NA |
| - Dietary fiber, g/d | 20.1 (0.6) | 22.7 (0.2) | 19.8 (0.3) | 20.8 (0.3) | NA |
| - Fruit and vegetables, g/d | 430 (24) | 506 (8) | 435 (11) | 501 (13) | NA |
|  |  |  |  |  |  |
| Including prevalent diabetics (n=32,430)^e^ |  |  |  |  |  |
| N (%) | 2530 (8) | 12630 (39) | 2496 (8) | 5383 (17) | 9391 (29) |
| Fruit, g/d | 215 (6) | 270 (3) | 229 (7) | 245 (4) | 273 (4) |
| Vegetables, g/d | 154 (4) | 173 (2) | 172 (4) | 176 (3) | 207 (2) |
| Dairy, g/d | 290 (8) | 267 (4) | 310 (10) | 254 (4) | 261 (4) |
| Fish, g/d | 10 (1) | 7 (0) | 7 (0) | 9 (0) | 9 (0) |
| Meat, g/d | 73 (1) | 81 (1) | 91 (2) | 74 (1) | 79 (1) |
| Oils and fats, g/d | 37 (1) | 33 (0) | 36 (1) | 38 (0) | 37 (0) |
| Sugary drinks, g/d | 97 (5) | 93 (2) | 92 (6) | 96 (3) | 87 (3) |
| Non-alcohol energy, kcal/d | 1638 (1) | 1717 (1) | 1736 (2) | 1637 (1) | 1727 (1) |
|  |  |  |  |  |  |
| Healthy Diet Indicator, score | 47.0 (0.3) | 47.3 (0.2) | 45.3 (0.3) | 48.1 (0.2) | 48.0 (0.2) |
| - Saturated fat, en% | 13.1 (0.1) | 12.6 (0.1) | 13.9 (0.1) | 12.8 (0.1) | 12.4 (0.1) |
| - PUFA, en% | 4.6 (0.1) | 5.7 (0.0) | 6.0 (0.1) | 4.9 (0.) | 5.6 (0.1) |
| - Protein, en% | 115.5 (0.1) | 16.3 (0.1) | 16.2 (0.1) | 15.6 (0.1) | 16.2 (0.1) |
| - Mono- and disaccharides, en% | 19.0 (0.2) | 19.7 (0.1) | 19.9 (0.3) | 18.6 (0.2) | 18.9 (0.1) |
| - Cholesterol, mg/d | 182 (2) | 201 (1) | 212 (3) | 182 (2) | 195 (2) |
| - Dietary fiber, g/d | 19.7 (0.2) | 20.8 (0.1) | 19.8 (0.2) | 19.7 (0.1) | 20.7 (0.1) |
| - Fruit and vegetables, g/d | 389 (9) | 481 (4) | 425 (9) | 454 (6) | 520 (6) |

Abbreviations: not applicable (NA); poly-unsaturated fatty acids (PUFA);

^a^ Adjusted for age, sex, education, employment, energy intake, smoking status, moderate alcohol consumption, and physical activity; ^b^ Adjusted for age, education, employment, self-reported prevalence of CHD or cancer, energy intake, smoking status, moderate alcohol consumption, and physical activity; ^c^ Adjusted for age, sex, education, employment, self-reported prevalence of CHD or cancer, energy intake, smoking status, moderate alcohol consumption, and physical activity; ^d^ Adjusted for age, sex, education, employment, self-reported prevalence of CHD or cancer, energy intake, smoking status, and physical activity;

^e^ Adjusted for age, education, employment, self-reported prevalence of CHD, cancer, or diabetes mellitus, energy intake, smoking status, moderate alcohol consumption, and physical activity.
